# Modelling Human Brucellosis Based on Infection Rate and Vaccination Coverage of Sheep and Goats

**DOI:** 10.3390/pathogens11020167

**Published:** 2022-01-27

**Authors:** Georgios Dougas, Aristomenis Katsiolis, Maria Linou, Polychronis Kostoulas, Charalambos Billinis

**Affiliations:** 1 Department of Microbiology, Medical School, National and Kapodistrian University of Athens, 11527 Athens, Greece; 2 General Veterinary Directorate, Hellenic Ministry of Rural Development and Food, 10176 Athens, Greece; arikavet27@yahoo.gr; 3 Hellenic Pasteur Institute, 11521 Athens, Greece; linoum@pasteur.gr; 4 Faculty of Public and One Health, University of Thessaly, 43100 Karditsa, Greece; pkost@uth.gr (P.K.); cbillinis@gmail.com (C.B.); 5 Faculty of Veterinary Science, University of Thessaly, 43100 Karditsa, Greece

**Keywords:** brucellosis, *Brucella melitensis*, vaccination, eradication, livestock

## Abstract

In this study, the vaccination coverage, serological sampling and infection rate of sheep and goats were evaluated as predictors for the modeling of human brucellosis in Greece. The human brucellosis disease frequency per local regional unit (RU) varied significantly (RR90) among consecutive years. The notification rate was higher (*p* < 0.001) in the RUs with implementation of vaccination in sheep and goats (vaccination zone—VZ) with a median of 1.4 (IQR 0.0–3.1) compared with the RUs of the eradication zone (EZ) with a median of 0.0 (IQR 0.0–0.0). In VZ, the increased frequency of human cases was associated with delayed vaccine administration (estimate: 0.14 (0.04; 0.29), *p* = 0.03) and higher vaccination coverage of the animals (estimate: −0.349 (−0.72; −0.07), *p* < 0.01). However, the flock sampling rate was highly heterogenous among RUs (IQR 10.56–52.93), and inconsistent within RUs throughout the period of the study 2013–2017 (*p* = 0.001), limiting the reliable estimation of the infection rate in livestock and the design of an integrated One Health model for human disease.

## 1. Introduction

Brucellosis is a debilitating zoonosis with worldwide occurrence [1]. *Brucella melitensis* is responsible for the vast majority of human cases [2]. Sheep and goats represent the natural hosts of *B. melitensis* [3,4]. The infection is transmitted to humans mainly by direct contact with animals or by consumption of milk products [5]. The disease is endemic in countries around the Mediterranean basin [6]. Greece presents the highest toll in human cases among European countries [7], with more than 90% of *Brucella* spp. isolates from clinical specimens identified as *B. melitensis* [8].

**Figure 1:**
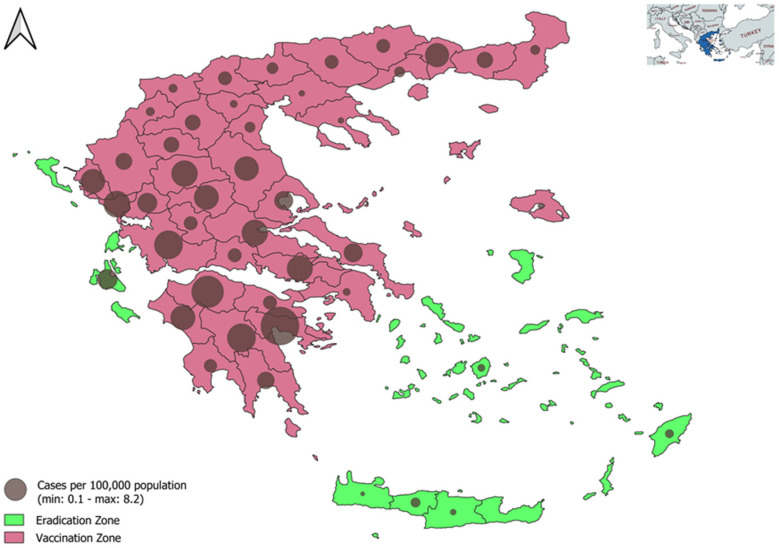
Mean annual brucellosis cases per 100,000 of human population, at local level (Regional Units), Greece, 2013–2017.

Since 1977, the basic concept for controlling and eradicating brucellosis in Greece has always been vaccination of herds combined with testing and slaughtering of seropositive animals. The State Veterinary Services from the Ministry of Rural Development & Food and the local Veterinary Services have implemented a control and eradication policy: in the vaccination zone (VZ), female animals are vaccinated, and males are blood-sampled, whereas in the eradication zone (EZ), a test-and-slaughter policy is applied (Figure 1). The transportation of animals from the VZ to the EZ is legally prohibited and in the VZ zone only vaccinated animals are allowed to move from one local Regional Unit (RU) to another. The VZ comprises the mainland and a few islands and has a population of 9,532,504 inhabitants (88.6% of the country’s population), whereas the EZ includes the main body of the islands with a population of 1,228,437 (11.4%) [9].

In the VZ zone, sheep and goats are vaccinated by the conjunctival administration of the attenuated live vaccine strain, Rev. 1, once in their life. The vaccine is administered only to females, ideally at the age of 3–6 months. The test-and-slaughter scheme is applied in the EZ and is based on the regular sampling and serological testing of all animals over the age of six months. Positive herds are identified by serology tests (Rose Bengal Test and Complement Fixation Test for screening and confirmation, respectively). Infected animals are slaughtered separately from healthy animals, taking all necessary biosecurity measures [10]. Unfortunately, this scheme did not result in eradication due to various reasons, and brucellosis remains endemic in livestock in Greece [11].

Agricultural professions and consumption of unpasteurized milk products are established risk factors for human brucellosis. Particularly, people having occupational contact with farm animals and their secretions or being involved in the processing of animal products, such as farmers, stockmen, shepherds, veterinarians, slaughtermen, and butchers, are at high risk of exposure to *Brucella* spp. [2,4]. However, despite the fact that sheep and goats are the reservoirs of the pathogen, specific markers have not been adequately described in livestock as predictors of human disease likelihood.

This study aimed primarily to assess the effect of infection rate, sampling intensity, and vaccination coverage of sheep and goats on the human brucellosis notification rate. Secondary objectives were to compare the frequency of human cases between VZ and EZ and to estimate the relative risk of brucellosis occurrence for RUs in Greece.

## 2. Results

### 2.1. Descriptive Results, Estimated Relative Risks and RR90

In Greece, in the period of the study (2013–2017), 621 human brucellosis cases were recorded and the notification rate per RU-year ranged from 0.00 to 12.3 with a median 0.7 (IQR 0.0–2.5). The notification rate in the VZ was significantly higher (*p* < 0.001), with a median 1.4 (IQR 0.0–3.1) compared with a median 0.0 (IQR 0.0–0.0) in the EZ (Figure 1).

The median age of cases was 47.0 years (IQR 32.3–58.0) and 69.6% were male. Among the cases, 77.6% (482/621) had a high-risk occupation or contact with farm animals.

A median of 85,219 (IQR 74,268–115,328) holdings and 14,502,751 (IQR 13,365,434–15,700,954) sheep and goats were recorded in Greece during 2013–2017. The average ratio of sheep to goats was 2.48 (SD = 0.10); however, data regarding the herd size and the composition of farmed species per holding were not available.

In the VZ, a median of 461 (IQR 294–804) unvaccinated holdings, 151,048 (IQR 118,370–240,229) unvaccinated sheep and goats and 6496 (IQR 2085–11,537) sheep and goats with delayed vaccination were identified annually, per RU, per 100,000 inhabitants (n = 195).

The rate of new infected flocks per RU and 100,000 inhabitants was higher in the VZ (mdn = 9, IQR 2–23) than in the EZ (mdn = 0, IQR 0–2) (*U* = 63.5, *p* = 0.00016) on an annual basis.

**Figure 2:**
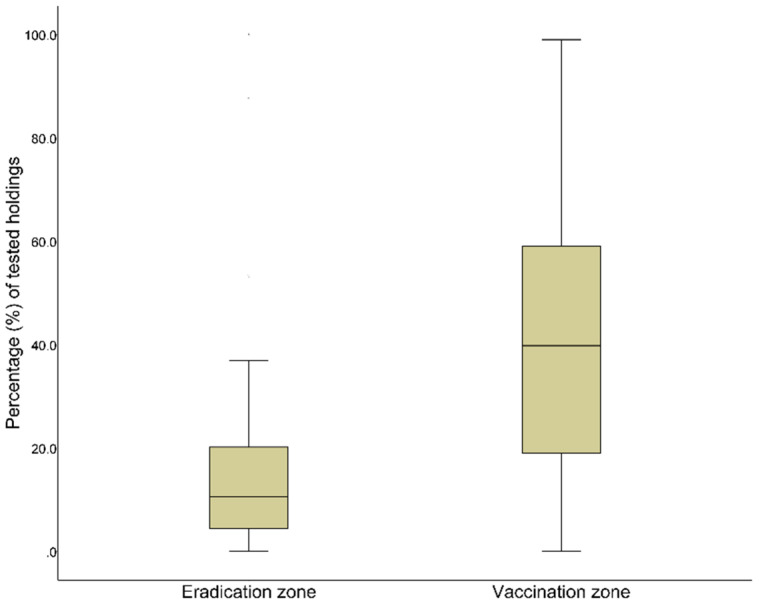
Percentage of tested holdings per Regional Unit on annual basis in the vaccination (n = 195) and eradication zone (n = 60), Greece, 2013–2017.

Among holdings, 22,899 (IQR 21,022–22,905) were annually tested for brucellosis. The sampling rates in all RUs of Greece throughout the study period varied from 0% to 100% with a median of 31.40% (IQR 10.56–52.93). The percentage of flock sampling per RU and year was significantly higher (*p* < 0.001) in the VZ, with a median of 39.8% (IQR 18.93–59.00) compared with 10.60% (IQR 4.35–20.23) in the EZ (Figure 2) (Supplementary Material: Table S1).

**Figure 3:**
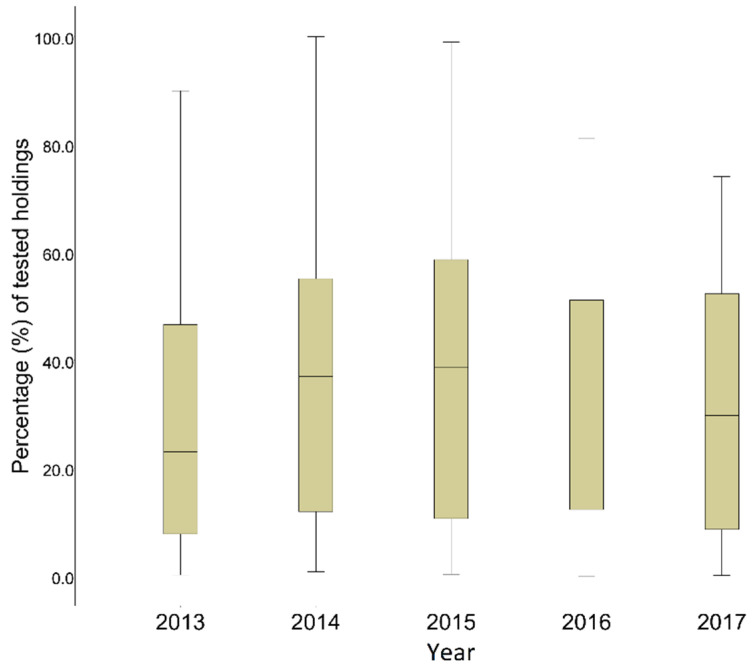
Median values and interquartile ranges of the annual sampling rates, Greece (n = 51), 2013–2017.

Nationwide sampling rates differed throughout the years, χ^2^(4) = 30.202, *p* < 0.0001 (Figure 3). Analysis, using Bayesian hierarchical Poisson models, revealed an increase in the sampling effort from 2013 to 2014 (Z = −3.828, *p* < 0.001) and to 2015 (Z = −3.374, *p* = 0.001) and a reduction in 2017 compared with 2015 (Z = −2.906, *p* = 0.004). A time effect during the years was also observed within RUs, Wilk’s lambda = 0.67, F (4, 46) = 5.659, *p* = 0.001, η^2^ = 0.33.

The newly diagnosed holdings with brucellosis had a positive correlation with the flock sampling rate on an annual basis (*r_s_*(208) = 0.565, *p*< 0.001).

Estimated relative risks (with 95% Probability intervals—PrIs) by each RU and year can be found in the Supplementary Material (Table S2). There was significant variability (RR90) in the human notification rate between consecutive years, as it was 7.998 (3.521; 20.31), 24.9 (5.287; 92.52), 13.84 (4.945; 41.17), 14.24 (4.443; 46.23) and 15.13 (4.661; 48.62) in the years from 2013 to 2017, respectively.

### 2.2. Effect of the National Brucellosis Program Parameters on the Number of Human Cases

Among the different hierarchical Poisson models, the model ignoring the temporal trends had the best fit to the data. Thus, the number of human cases in the previous year does not affect the number of observed cases in the current year. For all RUs in Greece (Model 1, Table 1), the number of human cases is positively associated with the application of a vaccination program. For RUs of VZ (Model 2, Table 2), the number of human cases is positively associated with the number of animals vaccinated at an age greater than 6 months per 100,000 humans but negatively associated with the number of the remaining unvaccinated animals per 100,000 humans.

**Figure 4:**
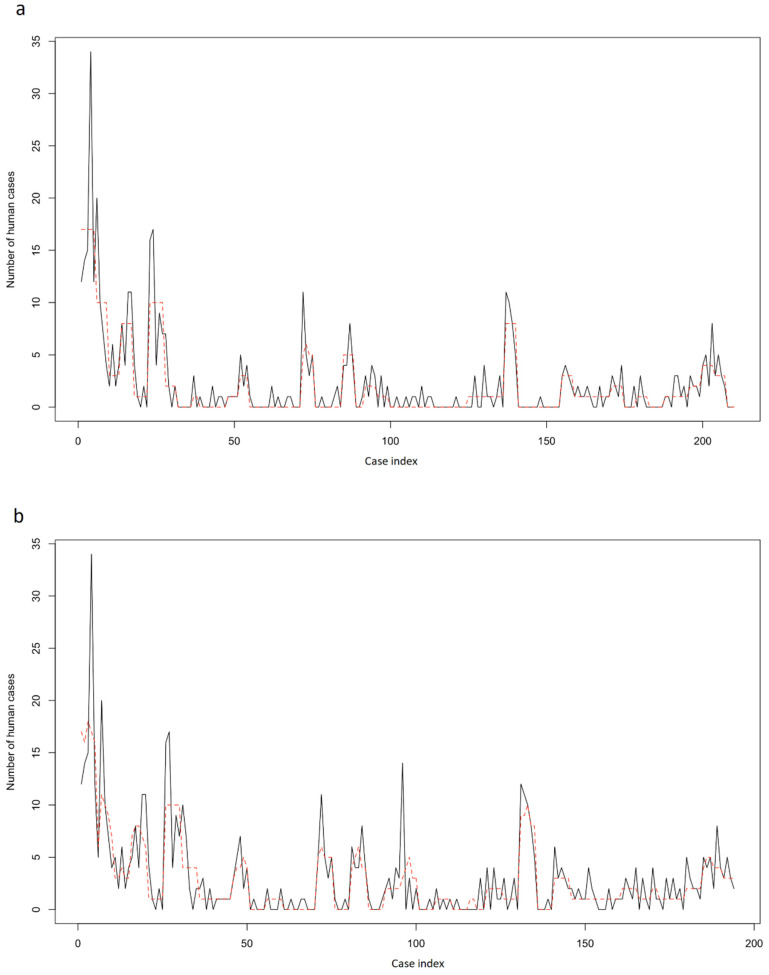
Observed (black line) and predicted (red dotted line) human brucellosis cases (**a**) under Model 1 in all Regional Units and (**b**) under Model 2 in the Regional Units of the vaccination zone, Greece, 2013–2017.

For both models 1 and 2, the plot of the predicted vs. the observed human brucellosis cases revealed an adequate fit of the final models to the data (Figure 4).

## 3. Discussion

This study examined the effect of sheep and goats’ brucellosis status, including vaccination, testing and infection rate, on the frequency of human brucellosis cases.

The data revealed a significant variability in the reported incidence of human brucellosis among RUs but also a lack of a consistent trend during consecutive years within RUs (as depicted by the RR90). This means that a very diversified setting exists in relation to the occurrence of the disease and the previous number of human cases does not affect present or future counts. The result is that a temporal trend does not exist at RU or country level.

Regions where vaccination is implemented presented a higher frequency of human brucellosis cases compared to the test-and-slaughter policy areas, in accordance with previous reports [12]. It is conceivable that the increased infection rate of the livestock in the VZ zone may lead to greater risk of human exposure.

Within RUs that vaccinate, the vaccination of female sheep and goats during the optimal age of three to six months had a protective effect for human brucellosis. This is in accordance with previous reports indicating that vaccination past the optimal age of 3–6 months fails to provide optimal protection to the animals and may cause increased spillover of *Brucella* spp. to the environment and higher likelihood of human exposure [13]. Furthermore, vaccinating within the optimal age may reflect adherence to the program and vaccinations performed according to the organized schedule. Vaccination in due time may also indicate increased compliance of the stockholder and closer veterinary attendance of the animals. The latter may aid in the early recognition of clinically suspect animals and application of biosecurity measures, therefore reducing the risk of infection [14]. High-risk practices can be avoided by adhering to safety protocols. In particular, the illegal exchange of sires among herds may increase the flock-to-flock dissemination of *B. melitensis*, as has been observed with other pathogens [15]. To our knowledge, there are no previous reports acknowledging the protective effect of the timely livestock vaccination on human brucellosis.

The study also revealed a seemingly paradoxical finding: a positive association of the animal vaccination coverage and the human disease rate. Vaccination was expected to have a protective effect against human disease as gaps in the vaccination program hinder eradication and maintain the disease in livestock [16]. We theorize that the veterinary authorities intensified the vaccination as a response to the locally increased flock infection rate or due to the higher number of human cases in the area.

The increased human brucellosis in areas where vaccination is more intensively implemented could theoretically be explained by the higher likelihood of accidental exposure to vaccine strains. The live attenuated *B. melitensis* vaccine (Rev. 1) may cause disease to humans indistinguishable from the one caused by the wild strains [17,18]. The inadequate use of personal protective equipment and the negligence to implement safe practices during high-risk activities such as vaccination, attending parturitions, handling aborted fetuses, and other obstetric procedures, increase the chance of infection [14]. In general, studies on the incidence of vaccine-induced human brucellosis, especially for the Rev. 1 vaccine, are scarce. Identification of wild or vaccine *Brucella* strains from clinical specimens is not routinely performed in Greece [19]; however, previous reports indicate that the vaccine strain can be held accountable for only a small proportion of human cases (0.65%, N = 308) [8]. A causative relation of human infection with the procedure of vaccinating the animals cannot be established based on the existing data.

Flock sampling was unexpectedly more intense in continental Greece than in the islands, even though a test-and-slaughter scheme is applied in the latter. Nationwide, sampling was highly variable among RUs, with a significant proportion of herds remaining untested. Our data indicated, as expected, that a higher flock sampling rate identified more infections. The weak and inconsistent sampling rates not only reduced the usefulness of infection data for modelling purposes, but also precluded the identification of recently infected herds. Undiagnosed new infections entail greater risk for humans than flocks with previously established infection due to a lack of awareness for biosecurity measures. Finally, a Bayesian model adequately fit human disease with delayed vaccination and vaccination coverage of the livestock. However, core predictors of the animal infection rate should be included in a cause-and-effect model. Good quality animal and human data are needed to explore the causalities among livestock and human brucellosis.

The underreporting from health settings across different RUs should also be considered in an integrated model of human disease. A nosocomial non-reporting rate of 24.1% to 35.0% of human brucellosis cases has been previously described in Greece [20].

Based on the findings of this study, the epidemiologic situation of human and animal brucellosis in Greece could be improved by implementing vaccination in due time and strengthening the serological screening; this may require allocating adequate resources to the local veterinary services and reassessing the efficacy of the existing brucellosis control and eradication program.

## 4. Materials and Methods

### 4.1. Data

Detailed data for brucellosis in sheep and goats for the years 2013 to 2017, stratified by RU and year, were obtained from the Department of Zoonoses of the Ministry of Rural Development & Food. The examined variables are shown in Table 3.

Human population data were extracted from the national census of 2011 (9 Hellenic Statistical Authority 2011).

Human brucellosis cases were defined according to European Commission decision 2018/945 requiring a combination of symptoms including fever, and laboratory findings (isolation of *Brucella* spp. by culture from a clinical specimen, and/or positive serological result with Standard Agglutination Test or Complement Fixation or ELISA, and/or detection of *Brucella* spp. DNA in a clinical specimen). Brucellosis is a mandatorily notified disease in Greece and cases are reported in the Greek National Public Health Organization (NPHO). Brucellosis notification rate represented the new human cases per RU, year, and 100,000 inhabitants and was derived from the regular data sharing between NPHO and the Department of Zoonoses of the Ministry of Rural Development & Food, and from the public database, available at the website of NPHO.

### 4.2. Bayesian Estimation of the Expected Number of Human Cases and the RU-Specific Relative Risk

Bayesian hierarchical Poisson models were used for the analysis.

We assume that the number of brucellosis cases in each of the *i*th RU follows a Poisson distribution:

$$y_{i}\sim Poisson\left(\lambda_{i}{Ε}_{i}\right)$$

where ${Ε}_{i}$ is the expected number of cases in each RU. Subsequently, a normal random effects prior is specified for $log\left(\lambda_{i}\right)$:

$$log\left(\lambda_{i}\right)=\alpha +\theta_{i}$$

with

α~Uniform(−∞,+∞)

$$\theta_{i}\sim Normal\left(0,\sigma^{2}\right)$$

$$\sigma^{-2}\sim Gamma\left(0.001,0.001\right)$$

α is the mean log relative risk, $\theta_{i}$ the random effects term for each RU and $\sigma^{2}$ the between RU variance, which captures the amount of extra-Poisson variation in the data. The $\theta_{i}$ can be considered as a latent variable, capturing the effects of unknown or unmeasured covariates that operate at the RU level. The relative risk ($RR_{i}$) in each of the RUs compared to the expected risk based on the average count of brucellosis cases in the whole country is calculated by:

$$RR_{i}=exp\left(\alpha +\theta_{i}\right)$$

Finally, the expected number of brucellosis cases per 10,000 population for each RU is calculated as:

$$cases_{i}=y_{i}^{rep}*10^{4}*n_{i}^{-1}$$

with $y_{i}^{rep}\sim Poisson\left(\lambda_{i}{Ε}_{i}\right)$ being the replicated counts for each RU under the fitted model and $n_{i}$ being the total population for each RU.

A useful summary metric for the variability among RUs, in a hierarchical setting, is the difference in the attained risk of brucellosis occurrence between the RUs with the higher and lower relative risk. If $\lambda_{5\%}$ and $\lambda_{95\%}$ denote the relative risk of brucellosis in the RU ranked at the 5th and 95th percentile, respectively, then RR90=λ5%λ5% is the ratio of the relative risks for brucellosis between the top and bottom 5% RUs.

### 4.3. Bayesian Assessment of the Effect of the Parameters of the National Brucellosis Program on the Number of Human Brucellosis Cases

As previously, let the number of human brucellosis cases $y_{i,j}$ for each of the ith years (with *i* = 2013, …, 2017) in each of the jth RUs follow a Poisson distribution:

$$y_{i,j}\sim Poisson\left(\lambda_{i,j}\right)$$

The logarithm for the mean $\lambda_{i,j}$ is modelled as,

$$log\left(\lambda_{i,j}\right)=a+X\beta +u_{j}$$

where *X* is the design matrix and encodes all known information about the independent variables and β is the (p×1) matrix for the coefficients corresponding to each of the *p* independent variables. We further considered two additional variations of this model in order to assess whether a calendar trend exists (i.e., the number of human cases in the previous year affects the number of observed cases in the current year). The first variation assumed a first order autoregressive structure (AR1):

$$log\left(\lambda_{i,j}\right)=a_{i}+X\beta +u_{j}$$

with

$$\alpha_{1}\sim Normal\left(0,\sigma^{2}/\left(1-\varphi_{1}^{2}\right)\right)$$

$$a_{i}|a_{1,\ldots ,i-1}=Normal\left(\varphi_{1}\alpha_{i-1},\sigma^{2}\right),\;i\geq 2$$

The second variation assumed a Normal random walk (RW1) prior for the $a_{i}$ coefficients:

$$a_{1}\sim Normal\left(0,0.001\right)$$

$$a_{i}\sim Normal\left(a_{i-1},1/\tau_{\alpha }\right),i\geq 2$$

Non-informative prior distributions were specified for $b\sim Normal\left(0,10^{3}\right)$ and the precision parameter $1/\tau_{\alpha }\sim Gamma\left(0.001,0.001\right)$. The autocorrelation parameter $\varphi_{1}$ was given a Uniform (−1,1) prior.

### 4.4. Selection of Independent Variables

We modelled the data in two steps and two separate sets of candidate variables were offered to each model. For the first model (Model 1), a set of variables that was recorded in all RUs was considered. These were: (i) vaccination (coded as yes or no for each RU), (ii) the total number of infected holdings per 100,000 citizens in the RU, (iii) the number of newly infected holdings per 100,000 citizens in the RU and (iv) the percent of tested holdings in the RU. The second model (Model 2) was relevant to the subset of data where a vaccination program was active. The variables offered to this model were (i) the remaining unvaccinated sheep and goats per 100,000 citizens in the RU, (ii) the remaining unvaccinated holdings per 100,000 citizens in the RU and (iii) the number of sheep and goats that were vaccinated after the age of 6 months (i.e., the optimal vaccination age is 3–6 months). Each of the two sets was offered independently to all models described in the previous section, and a backward selection procedure was repeated until all remaining variables were significant at *p* < 0.05. Bayesian p-values were calculated using the step function in OpenBUGS [21].

### 4.5. Model Selection Criteria and Model Goodness-of-Fit Tests, Selection of the Final Model and Assessment of the Adequate Fit to the Data

Selection of the best model was based on the comparison of the deviance information criterion (DIC), which is a global measure of comparative model fit [22]. The model with the lowest DIC was chosen. Yet, DIC and other similar indices are measures of comparative model fit and indicate which model best fits the data at hand. However, they do not assess whether the best model has an adequate fit to the data. This can be evaluated by the use of goodness-of-fit tests, which are based on the comparison between the observed data and the predictions under the model [23].

### 4.6. Convergence Diagnostics and Software

The convergence check for the MCMC chain was based on a combination of checks because convergence diagnostics of the MCMC chain are not foolproof. Therefore, a combination of the Raftery and Lewis method, the Gelman–Rubin diagnostic [24,25] autocorrelation checks and visual inspection of the trace plots and summary statistics was used as recommended by [26]. Parameter estimates were based on analytical summaries of 49,500 iterations of three chains after a burn-in phase of 500 iterations. This was adequate because the Raftery and Lewis method suggested that analytical summaries of 45,000 iterations after a burn-in of 15 iterations were needed. All checks suggested that convergence occurred and autocorrelations dropped off quickly. Models were run in the freeware program OpenBUGS [21] and the code is available as upon request from PK.

## 5. Conclusions

Delayed vaccination of sheep and goats, past the optimal age of 3–6 months, was associated with a higher human brucellosis disease rate.

A positive association of livestock vaccination with human brucellosis was explained as the result of an enhanced veterinary response to increased animal or human infection.

Strengthening of veterinary sampling is crucial for a reliable estimation of infection rate and the development of integrated models of human brucellosis.

## Figures and Tables

**Table 1:** Parameter estimates (means and 95% probability intervals—PrIs) of the fitted model for all Regional Units in Greece (model 1).

Variable		Estimate	*p*
Intercept		−1.84 (−2.81; −0.95)	<0.01
Vaccination	No	Reference	
	Yes	2.31 (1.35; 3.32)	<0.01
Random-effect variance		1.23 (0.88; 2.22)	<0.01

**Table 2:** Parameter estimates (means and PrIs) of the fitted model for the Greek Regional Units where a vaccination program is applied (model 2).

Variable	Estimate	*p*
Intercept	0.56 (0.17; 0.94)	<0.01
Remaining unvaccinated female animals per 10^5^ humans	−0.349 (−0.72; −0.07)	<0.01
Female animals vaccinated at age > 6 mo per 10^5^ humans	0.14 (0.04; 0.29)	0.03
Random-effect variance	1.23 (0.72; 2.20)	<0.01

**Table 3:** Sheep and goats’ husbandry and brucellosis status data investigated for association with human brucellosis.

Variable	Definition
“New positive holdings” ^a^	Only newly diagnosed with brucellosis holdings in a particular year
“Unvaccinated animals” ^a^	Female sheep and goats remaining unvaccinated
“Unvaccinated holdings” ^a^	Farms with sheep and/or goats, in which no vaccination was implemented in the animals eligible for vaccination
“Animals with delayed vaccination” ^a^	Female sheep and goats that received vaccination after the optimal age (3–6 months old)
“Percentage of tested holdings”	Percentage of holdings tested serologically

^a^ per Regional Unit, year, and 100,000 of human population.

## Data Availability

Data are publicly available at https://doi.org/10.5281/zenodo.5806444, accessed on 27 December 2021.

## Supplementary Materials

The following supporting information can be downloaded at: https://doi.org/10.5281/zenodo.5806444, accessed on 27 December 2021, Table S1: Brucellosis in Humans, Sheep and Goat Farms, Table S2: Relative risks (with 95% Probability intervals—PrIs) by Regional Unit (RU) and year.
